# Microstructure and optical properties of nanocrystalline Cu_2_O thin films prepared by electrodeposition

**DOI:** 10.1186/1556-276X-9-219

**Published:** 2014-05-07

**Authors:** Xishun Jiang, Miao Zhang, Shiwei Shi, Gang He, Xueping Song, Zhaoqi Sun

**Affiliations:** 1 School of Physics and Material Science, Anhui University, Hefei 230601, China; 2 School of Mechanical and Electronic Engineering, Chuzhou University, Chuzhou 239000, China

**Keywords:** Cu_2_O films, Microstructure, Morphology, Optical properties

## Abstract

Cuprous oxide (Cu_2_O) thin films were prepared by using electrodeposition technique at different applied potentials (−0.1, −0.3, −0.5, −0.7, and −0.9 V) and were annealed in vacuum at a temperature of 100°C for 1 h. Microstructure and optical properties of these films have been investigated by X-ray diffractometer (XRD), field-emission scanning electron microscope (SEM), UV-visible (vis) spectrophotometer, and fluorescence spectrophotometer. The morphology of these films varies obviously at different applied potentials. Analyses from these characterizations have confirmed that these films are composed of regular, well-faceted, polyhedral crystallites. UV–vis absorption spectra measurements have shown apparent shift in optical band gap from 1.69 to 2.03 eV as the applied potential becomes more cathodic. The emission of FL spectra at 603 nm may be assigned as the near band-edge emission.

## Background

Known as a p-type semiconductor, cuprous oxide (Cu_2_O) has the advantages of low consumption, nontoxic, and higher conversion efficiency. Therefore, it is widely used in solar cells, lithium ion batteries, biological sensors, gas sensors, magnetic storage, microdevices, and negative electrodes [1-6]. The good electro-optical properties of Cu_2_O make it used as photocatalyst in degradation of organic pollutants and H_2_ evolution from photoelectrolysis of water under visible light illumination [7-9]. By far, many deposited methods have been investigated to prepare Cu_2_O thin films, such as sputtering [10,11], thermal oxidation [12], chemical vapor deposition [13], anodic oxidation [14], spray pyrolysis [15,16], chemical oxidation [17], electrodeposition [18,19], and so on. Among these techniques, electrodeposition is an inexpensive, convenient, and effective way to prepare semiconductor oxide films over conductive substrates. The surface morphology and physical properties of the electrodeposition-derived films is mainly determined by deposition parameters such as applied potential, concentration of electrolyte, bath temperature, and bath pH [20-23].

Yao et al. [24] reported the electrochemical deposition of Cu_2_O microcrystals on a glassy carbon (GC) electrode. When varying the deposition voltage at GC electrode, Cu_2_O nanocrystalline changed from superoctahedral to octahedron and then to microspheres. Jiang et al. [25] studied electronic structure of Cu_2_O thin films grown on Cu (110) by X-ray absorption spectroscopy (XAS) and X-ray photoelectron spectroscopy (XPS). Combined with XAS and XPS measurements, accurate identification of the various chemical components has been determined.

According to these observations, it can be concluded that the deposition conditions play an important role in the physical properties of Cu_2_O thin films. And they also explained about the effect of deposition conditions on the microstructure and optical properties of Cu_2_O films. Recently, the electrodeposited Cu_2_O films prepared using potentiostatic method and physical properties of the as-deposited Cu_2_O films have been reported. In this paper, Cu_2_O thin films were deposited by electrodeposition at different applied potentials. The effect of the applied potential on the morphological, microstructural, and optical properties of the as-deposited Cu_2_O films has been investigated in detail.

## Methods

### Preparation of Cu_2_O thin films

The Cu_2_O thin films were prepared by electrodeposition on Ti sheets. Prior to the deposition, Ti sheets were ultrasonically cleaned in acetone, alcohol, and deionized water, sequentially. Then, they were chemically polished by immersing them in a mixture of HF and HNO_3_ acids (HF:HNO_3_:H_2_O = 1:1:2 in volume) for 20 s, followed by rinsing in deionized water.

Electrodeposition of Cu_2_O was performed using a three-electrode system, in which a Ti sheet was used as a working electrode. A Pt plate and an Ag/AgCl in saturated potassium chloride aqueous solution were employed as counter and reference electrode. Cu_2_O films were grown on the surface of Ti sheets at bath temperature of 40°C using a solution consisting of 0.1 M sodium acetate (NaCH_3_COO) and 0.05 M cupric acetate (Cu(CH_3_COO)_2_). Electrodeposition was carried out under potentiostatic condition at different applied potentials (−0.1, −0.3, −0.5, −0.7, and −0.9 V) with respect to the reference electrode. The five samples were denoted as S1, S2, S3, S4, and S5, respectively. Finally, the obtained samples were annealed in vacuum at a temperature of 100°C for 1 h.

### Characterization

The surface morphology of the electrodeposited films was examined by field-emission scanning electron microscope (SEM, Hitachi, S4800, Tokyo, Japan). To determine the phase and crystalline structure of the as-deposited films, X-ray diffraction (XRD, MAC Science, Yokohama, Japan) analysis was carried out with an X-ray diffractometer employing Cu-K_α_ radiation. The UV-visible (vis) absorption spectra were recorded by a UV–vis spectrometer (Shimadzu, UV-2550, Kyoto, Japan). The FL spectra of the films were examined by a fluorescence spectrometer (Hitachi Corp., FL-4500).

## Results and discussion

### Structural characterization

**Figure 1:**
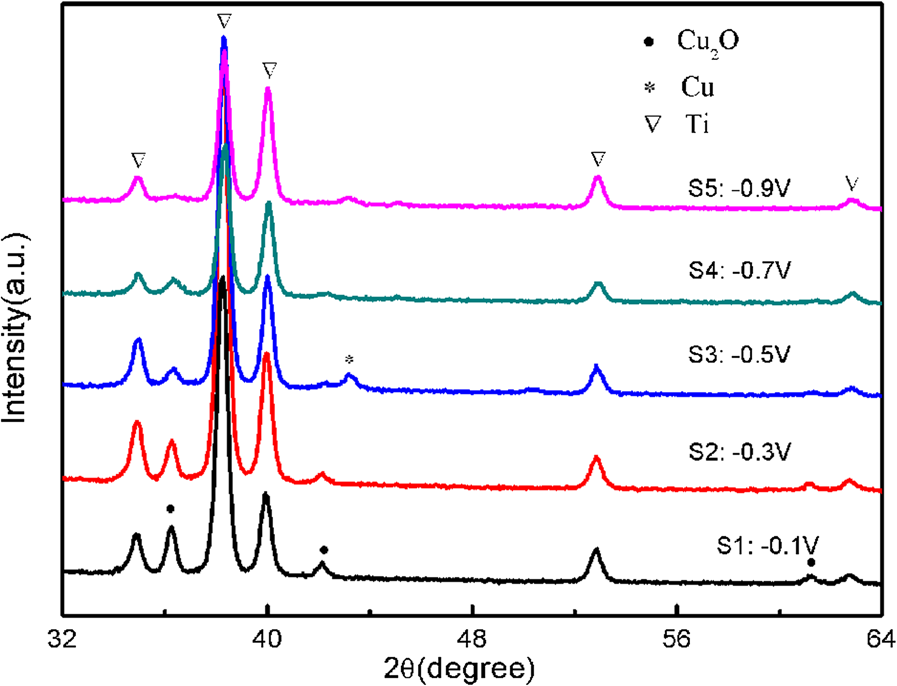
**X-ray diffraction patterns for the Cu**_**2**_**O films.**

Figure 1 illustrates the XRD profiles of the Cu_2_O films deposited at applied potentials between −0.1 and −0.9 V vs. the reference electrode.

Apart from the diffraction peaks corresponding to the Ti sheet, the peaks with 2*θ* values of 36.28°, 42.12°, and 61.12° corresponding to (111), (200), and (220) crystal planes, respectively, are assigned as the pure Cu_2_O (JCPDS: 05–0667). When deposition is carried out at −0.5 V, the peak of Cu is observed, suggesting that some metal copper form in the electrodeposition process [26].

Based on Figure 1, it can be noted that the intensity of Cu_2_O peaks decrease with increasing the deposition potential. Peaks corresponding to the Cu_2_O disappear when deposited at −0.9 V. This may be due to quicker growth of Cu_2_O particles and worse crystallization at higher applied potential.

### Surface morphology

**Figure 2:**
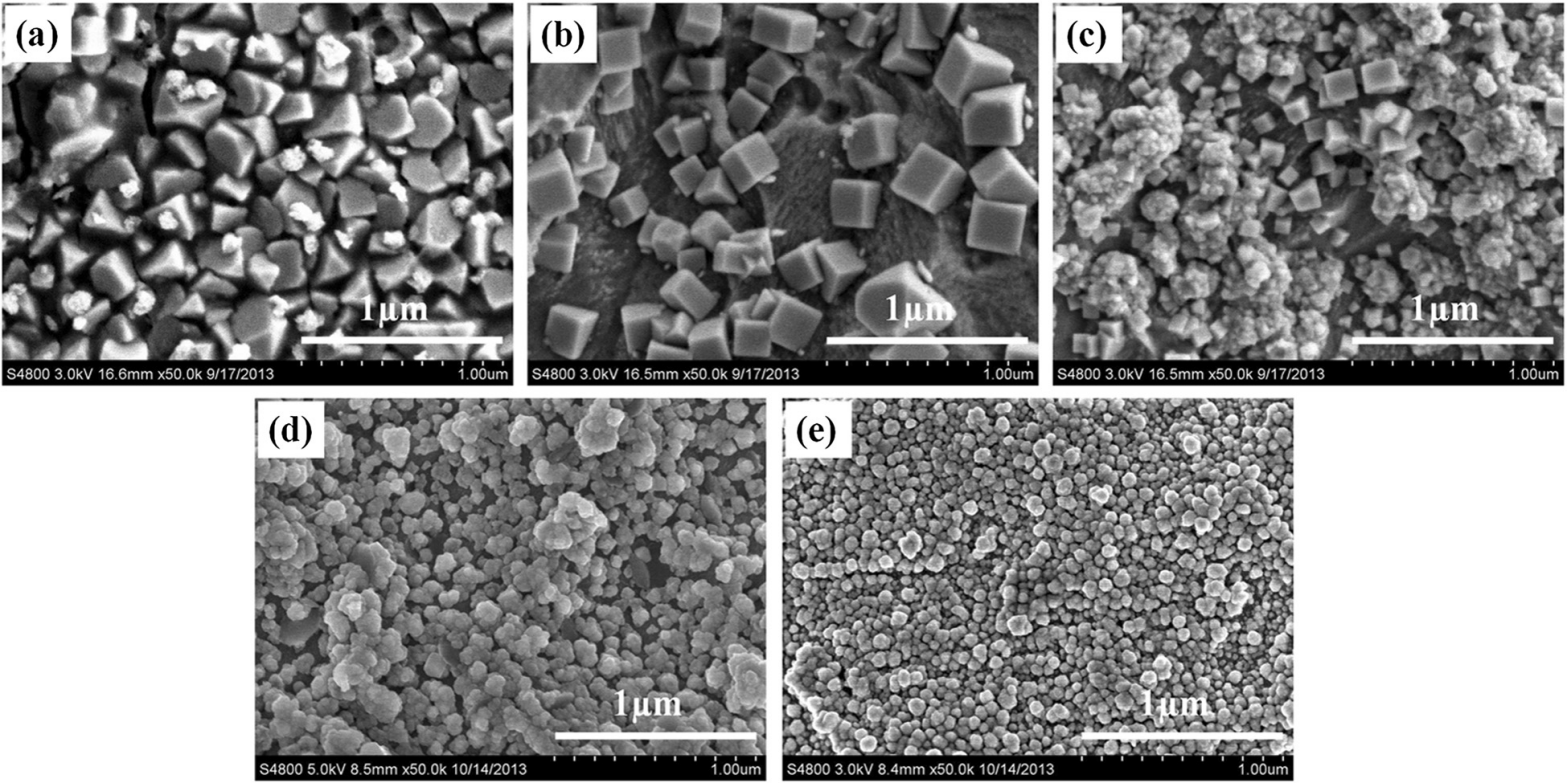
**SEM micrographs of Cu**_**2**_**O films. (a)** −0.1 V, **(b)** −0.3 V, **(c)** −0.5 V, **(d)** −0.7 V, and **(e)** −0.9 V.

The SEM micrographs of the Cu_2_O films deposited at different applied potentials are shown in Figure 2. The morphology of the Cu_2_O particles changes obviously with increasing the applied potential. The films deposited at −0.1, −0.3, and −0.5 V vs. the reference electrode (Figure 2a,b,c, respectively) are formed by regular, well-faceted, polyhedral crystallites. The films change from octahedral to cubic and then to agglomerate as the applied potential becomes more cathodic.

From Figure 2, it can be observed that the Cu_2_O thin film deposited at −0.1 V vs. the reference electrode exhibits pyramid shaped structure, as shown in Figure 2a, whereas the film deposited at −0.3 V exhibits cubic structure (Figure 2b). Cuprous oxide (111) crystal plane has the highest density of oxygen atoms, and the growth rate is smaller at lower deposition potential. So morphology of Cu_2_O films depends on (111) crystal plane, leading crystal surface morphology to pyramid with four facets (Figure 2a). Figure 2c shows co-deposition of agglomerate Cu with cubic structure Cu_2_O when deposited at −0.5 V, which is in good agreement with the observation confirmed by XRD spectra shown in Figure 1.

Figure 2d,e shows the SEM micrographs of films deposited at −0.7 and −0.9 V vs. the reference electrode, respectively. These films exhibit a granular spherical morphology, and the average diameter of the grains tends to be approximately 50 nm.

### Optical properties

**Figure 3:**
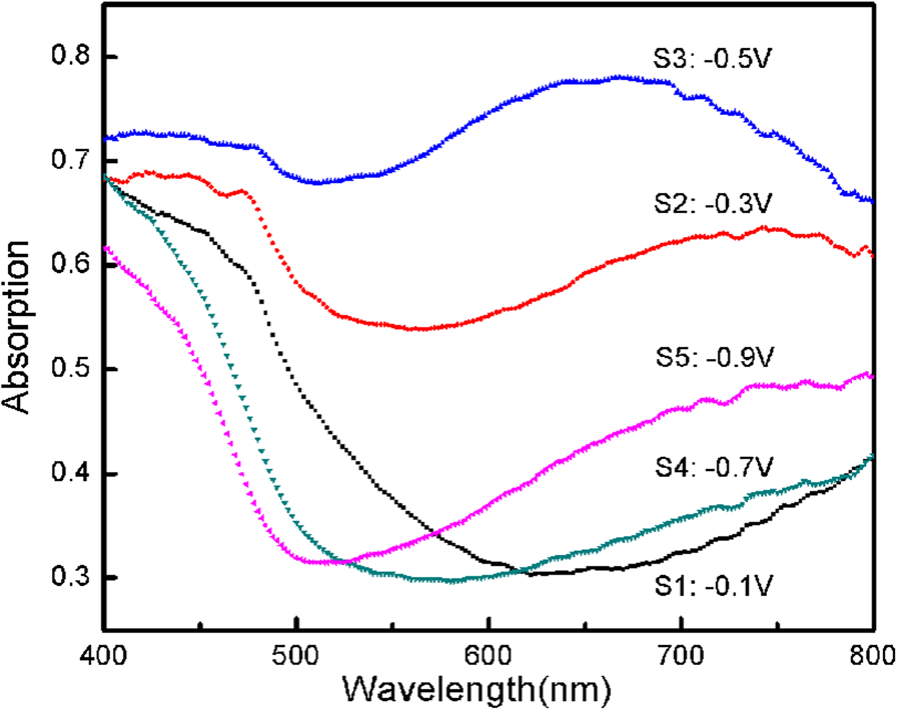
**UV–vis absorption spectra of Cu**_**2**_**O thin films.**

Figure 3 illustrates the optical absorption spectra for all the samples of cuprous oxide thin films deposited on Ti sheets at different applied potentials. As can be seen, there is an absorption edge in the range of 500 to 620 nm. Comparing these curves, it can be found that the absorption edges show redshift then blueshift with increasing the applied potential.

The photoabsorption in the visible light range for Cu_2_O film at −0.1 V vs. the reference electrode with cubic structure was more than 50% stronger than that for Cu_2_O film with pyramid shaped structure, which can be seen from Figure 2a,b. It can originate from the reason that the cubic structure film has more surfaces to adsorb light, leading to stronger photoabsorption [27].

Cu_2_O film deposited at −0.5 V vs. the reference electrode with the strongest absorption is due to the resonance absorption of metal copper particles, which can be also confirmed by XRD spectra of Figure 1. The decrease of the absorption coefficient of Cu_2_O films deposited at −0.7 and −0.9 V may be due to too much nucleation covering the entire Ti sheets. It decreases gaps, and defects of the films then reduce the scattering of light.

**Figure 4:**
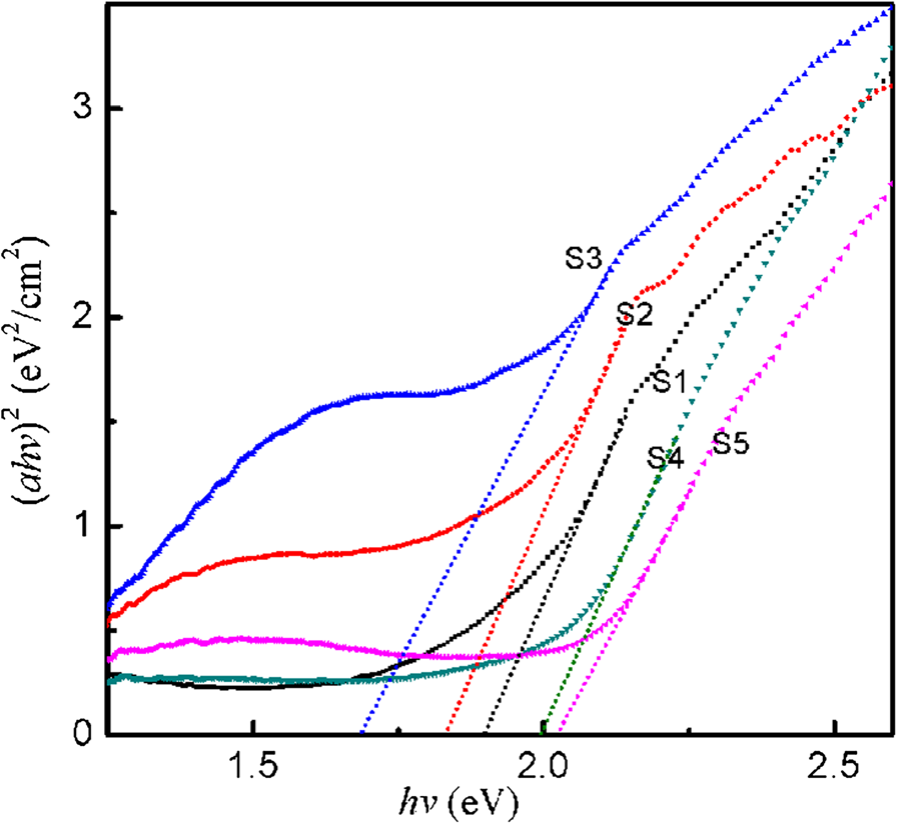
**Square of the absorption energy as a function of photon energy of Cu**_**2**_**O films.**

The cuprous oxide is a typical direct band gap semiconductor. The absorption coefficient satisfies the equation (*ahv*)^2^ = *A*(*hv* − *E*_*g*_) for a direct band gap material [28]. Here, *a* is the absorption coefficient, *A* is a constant, *hv* is the discrete photon energy, and *E*_*g*_ is the band gap energy. The band gap *E*_*g*_ is obtained by extrapolation of the plot of (*ahv*)^2^ vs. *hv*, and the estimated direct band gaps of Cu_2_O films are listed in Table 1. Based on the data of Figure 4 and Table 1, it can be found that the band gap of Cu_2_O films first decreases and then increases with the applied potential which becomes more cathodic. The intercepts to the (*ahv*)^2^ vs. *hv* plot for the samples S1 and S2 give the value of band gap as 1.90 and 1.83 eV, respectively. Due to the presence of metal Cu particles, the absorption edge of the sample S3 is 1.69 eV. Figure 4 shows (*ahv*)^2^ vs. *hv* plot for the samples S4 and S5, and the obtained band gap values are 2.00 and 2.03 eV, respectively. This is also consistent with previous XRD results and coincides with Grez's observation [29].

**Table 1:** **The estimated direct band gaps of Cu**_**2**_**O films**

**Applied potential (V)**	**−0.1**	**−0.3**	**−0.5**	**−0.7**	**−0.9**
Band gap (eV)	1.90	1.83	1.69	2.00	2.03

**Figure 5:**
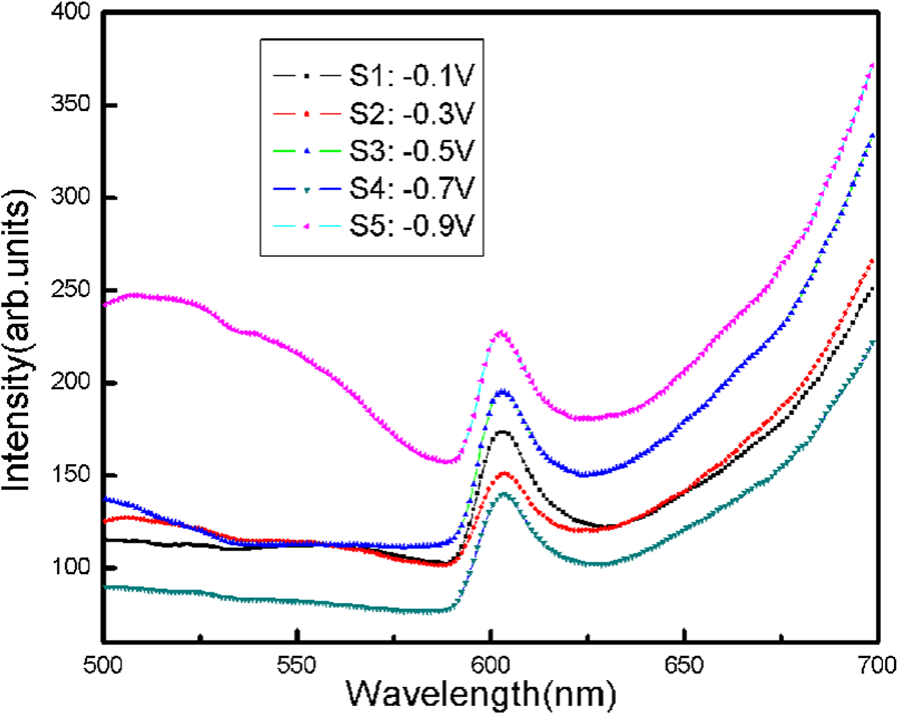
**FL spectra of Cu**_**2**_**O thin films.**

It is well known that FL spectra of semiconductor materials can be introduced to provide some information about the structure of energy band and the crystalline quality. In this work, the excitation wavelength of 400 nm is used as the excitation source with photon of 3.10 eV, which is higher than the band gap of Cu_2_O. Room temperature FL spectra results for samples deposited at the different applied potentials are individually presented in Figure 5. The FL signals of the samples are quite similar. The primary FL spectral characteristics for all samples include an emission peak centering at about 603 nm (2.06 eV). As the band gap of Cu_2_O is about 2.0 eV, the emission at 603 nm can be attributed to near band-edge emission from free exciton recombination [30].

## Conclusions

In summary, Cu_2_O thin films were deposited on Ti sheets in a solution consisting of cupric acetate and sodium acetate by electrodeposition method. XRD measurement shows the existence of Cu_2_O with cubic structure and the peak of Cu only at −0.5 V. SEM images reveal that the applied potential has significant influence on the surface morphology. The morphology of Cu_2_O films turns octahedral into cubic and agglomerate as the applied potential becomes more cathodic. Band gap values of the films vary from 1.83 to 2.03 eV. The emission at 603 nm (2.06 eV) of FL spectra can be caused by near band-edge emission from free exciton recombination.

## Competing interests

The authors declare that they have no competing interests.

## Authors’ contributions

XSJ and MZ prepared the films and tested the surface topography. X-ray diffraction was investigated by SWS and XPS. The surface morphology and optical properties were measured by GH and ZQS. The calculations were carried out by XSJ who also wrote the manuscript. Besides, MZ helped to draft the manuscript. All authors read and approved the final manuscript.
